# Emergence and Mechanism of Resistance of Tulathromycin Against *Mycoplasma hyopneumoniae* in a PK/PD Model and the Fitness Costs of 23S rRNA Mutants

**DOI:** 10.3389/fvets.2022.801800

**Published:** 2022-02-11

**Authors:** Xirui Xia, Lan Yang, Yuzhou Ling, Jiao Yu, Huanzhong Ding

**Affiliations:** Guangdong Key Laboratory for Veterinary Drug Development and Safety Evaluation (SCAU), South China Agricultural University, Guangzhou, China

**Keywords:** *Mycoplasma hyopneumoniae*, pharmacokinetic/pharmacodynamic, tulathromycin, 23S rRNA, fitness cost

## Abstract

Macrolides are widely used in diseases caused by *Mycoplasma* spp. The new semi-synthetic macrolide antibiotic tulathromycin is currently in wide use for the treatment of respiratory diseases of livestock. The objective of this study was to evaluate the antibacterial effect of tulathromycin against *Mycoplasma hyopneumoniae* using an *in vitro* pharmacokinetic/pharmacodynamic (PK/PD) model to reveal mechanisms of antibiotic resistance and to evaluate the fitness of drug-resistant strains. In this study, high performance liquid chromatography-tandem mass spectrometry was used to determine drug concentrations for the *in vitro* model after dosing. The peak concentrations were in the range 0.3125–20 μg/mL (1 × MIC-64 × MIC). The ratio of the area under the concentration-time curve (AUC) over 72 h divided by the MIC (AUC_72h_/MIC) had the highest correlation with the antibacterial effect of tulathromycin against *M. hyopneumoniae*. Tulathromycin also showed concentration-dependent antimicrobial effects and promoted the emergence of drug-resistant bacteria after being cultured for 168 h and most were mutations in 23S rRNA at site A2058G (*E.coli* numbering) and only a single isolate was an A2058T (*E.coli* numbering) mutant. In the presence of reserpine, we determined the MIC of tulathromycin, tilmicosin, tiamulin and tylosin against these drug-resistant bacteria and the strains with efflux pump mechanisms were found among the strains resistant to tilmicosin. Gene expression analysis indicated that the ABC and MATE transporter efflux pump genes RS01935, RS02670, RS01115, RS01970, RS02395 and RS03540 (MATE family efflux transporter) were up-regulated in the three strains (*P* < 0.05 or *P* < 0.01). These investigations provide guidance for clinical administration of tulathromycin and elucidate the mechanism and fitness cost of drug resistance in *M. hyopneumoniae*.

## Introduction

*Mycoplasma hyopneumoniae* is the primary pathogen of endemic pneumonia in pigs and has a high incidence leading to epidemics. *M. hyopneumoniae* parasitizes the trachea and bronchiolar epithelial cells and interferes with normal cilia functions leading to retention of lung secretions causing difficulty breathing, coughing, loss of appetite and weight loss (1). Secondary infections by *Pasteurella* spp. and *Actinobacillus pleuropneumoniae* are common in pigs affected at this stage (2). Treatment of these infections are problematic because of the unique cell membrane structure of *M. hyopneumoniae* and antibiotics that inhibit folate metabolism are ineffective. In contrast, pleuromutilins, fluoroquinolones, macrolides and tetracyclines have good antibacterial effects (3, 4). In particular, pleuromutilins and macrolides inhibit the action of peptidyl transferase and inhibit protein synthesis by binding to the 23S rRNA domain V (5, 6).

The macrolide tulathromycin has a short half-life of absorption and a long half-life of elimination and has been used to treat mycoplasma infections. It can rapidly reach the maximum concentration (*C*_max_) and maintain a high concentration in plasma (7, 8). Mutations in 23S rRNA and active drug efflux are the primary mechanisms of resistance to the macrolides. In *Aeromonas hydrophila*, inactivation of the TonB system significantly compromises the resistance to macrolides, and the mechanism of action is related to the function of MacA2B2-mediated macrolide efflux. MacA2B2 is one of two ATP-binding cassette (ABC) types of the macrolide efflux pump (9). Through genome-wide analysis, LI Shao LI et al. found that in macrolidene-resistant *M. pneumoniae*, 4 SNPs were clustered in macB, a gene encoding ATP binding cassette transporter family (10, 11). Efflux is primarily mediated by ATP-binding cassette (ABC) and the Multidrug and Toxic Compound Extrusion (MATE) family transporters (12). It has been suggested that the resistance of *M. hominis* to ciprofloxacin is the result of the efflux transporters of MATE family. There are only a few reports that such efflux pumps can transport macrolides (13, 14).

Reserpine is a kind of plant extracts, an indole alkaloid present in a variety of *Rauwolfia serpentina* plants. It has an inhibitory effect on many types of efflux pumps, Including ABC transporters (15). Reserpine reverses drug resistance in a variety of bacteria mediated by the efflux pump (16, 17). *M. hominis* uptake of fluoroquinolones was significantly reduced in the presence of reserpine or orthovanadate. This demonstrated that fluoroquinolone drug resistance was related to active efflux. In addition, the active efflux for *M. hominis* is primarily the result of ABC proteins (18). In this study, reserpine was used to test whether the macrolides resistance of *M. hyopneumoniae* was related to the efflux pump mechanism.

The primary mutations associated with the acquisition of resistance are A2058G and A2058C for *M. hyopneumoniae* and C2057A, C2610U and A2059G mutations for *Mycoplasma hominis* (10, 19, 20). Although tulathromycin, tilmicosin and tylosin are macrolides, their structures are different. Tilmicosin and tylosin are 16-membered macrolides antibiotics with similar structures that can bind directly to site A2058 in the V region of bacterial 23S rRNA (21). The main component of tulathromycin is a 15-membered macrolide agent, whose molecular structure is shown in the reference. There is no report on the ribosomal binding of tulathromycin. Whether the 23S rRNA mutation induced by this drug is the same as that induced by cetacyclic macrolides is unknown (22). Tiamulin belongs to pleuromutilin antibiotics. Although its antibacterial mechanism is similar to macrolides, its binding situation on 23sRNA is completely different from that of macrolides. Drug molecules cannot directly bind to site A2058, and there is no common binding site between the two drugs (23).

Although 23S RNA mutations are genomic mutations that can only be inherited vertically and cannot be transmitted horizontally, the risk of drug resistance still exists. If a mutant is stable in an antibiotic-free environment and is better adapted than the parent strain, it is likely to gradually replace the sensitive strain in a farmed environment.

Bacterial adaptability during commensal life as well as infections includes the ability to grow and invade the animal body. Drug target sites in microorganisms are most often linked with essential life activities and target site mutations in addition to resistance can also adversely affect the physiological activities of the bacteria and a resulting cost in fitness (24, 25). For instance, rpoB mutations in *Riemerella anatipestifer* mediate rifampin resistance at a cost to its RNA polymerase activity (26). The A2074C mutation in 23S rRNA of *Campylobacter jejuni* caused more severe fitness costs due to its more proximal linkage with translation (27).

Pharmacokinetic/pharmacodynamic (PK/PD) model studies primarily are focused on relationships between pharmacokinetic parameters and antibacterial effects and not with enrichment of drug-resistant bacteria. Additionally, the drug studies that result in the sequence analysis of drug resistance determinants have not evaluated fitness costs.

The fitness costs that drug-resistant bacteria develop during their development of resistance also play a role in bacterial responses to drug resistance and can be seen as a liability or risk for these organisms. We applied this logic to different drug resistant populations of *M. hyopneumoniae* and estimated the PK parameters along with drug-resistance bacterial variables. During the operation of the model, we also monitored the changes in the number of drug-resistant bacteria to clarify relationships between PK and enrichment of drug-resistance. The drug resistant determination region of resistant bacteria was amplified and sequenced and efflux pump inhibition tests were carried out and verified using real-time PCR. We used this information to assess the mechanisms that these mutants display when they are confronted with dynamic alterations in drug concentrations and to assess whether the mutants developed a competitive advantage over the parental strain. We compared resistant bacteria from different drugs in order to identify the mutation types that were most adaptable and had the greatest risk of transmission. This is helpful to evaluate the risk of drug resistance and to screen out antimicrobial agents that are less likely to cause drug resistance.

## Materials and Methods

### Bacterial Strains and Antibiotics

The *M. hyopneumoniae* standard strain ATCC 25934 was acquired from the Chinese Veterinary Microorganism Culture Collection Center (Beijing, China). Strains MTIL1~3 MTIA1~2 were isolated in previous study from *in vitro* selection using a dynamic model and were ATCC 25934 siblings (19, 20). MTUL1~2 were isolated in this study. Interestingly, the A2058C mutant reported in previous studies returned to wild type with passage and could not be used for subsequent experiments. We re-measured the MIC of all strains isolated at that time and sequenced, and finally found the A2058T mutants MTIA1 and MTIA2. All *M. hyopneumoniae* cultures were preserved by storage at −80°C. All strains were cultured at 37°C in the presence of 5% in pig mycoplasma culture medium containing swine serum, NADH and L-cysteine cysteine were purchased from Qingdao Hope Biological Technology (Qingdao, China). Tilmicosin 75.8%, tiamulin 99.0% and tylosin 82.6% were purchased from Guangdong Dahuanong Animal Health Products (Guangdong, China). Tulathromycin 98.8% was provided by Shandong Lukang Shelile Pharmaceutical (Shandong, China).

### Minimum Inhibitory Concentration, the Minimum Concentration Inhibiting Colony Formation by 99% (MIC99) and Mutant Prevention Concentration Assays

The MIC values of antibiotics against *M. hyopneumoniae* was determined using both the standard micro-dilution and agar-dilution methods as described previously (19, 28). The standard method used *M. hyopneumoniae* cultures in the exponential growth phase period at 10^6^ CFU/mL that was added at 100 μL per well in 96-well cell culture plates containing a series of equal volumes of test drug, growth control (medium only), end point control (blank medium) and sterile control (blank broth). The plate was sealed and cultured as per above and color development was observed every 24 h and when the positive control changed from pink to orange, the minimum drug concentration whose color was consistent with the negative control was taken as the MIC result. The acid produced by *M. hyopneumoniae* as it grows changes the color of the medium from red to orange, which was used as the basis for interpreting MIC results.

The agar dilution method (29) consisted of agar plates containing a two-fold dilution series of antibiotic concentrations and 10 μL of culture medium (see above) in triplicate were then placed on the surface and the plates were incubated as per above for 7 d. The lowest drug concentration in the drug-containing medium lacking growth was recorded as the MIC as observed using an inverted microscope.

MIC_99_: MIC_99_ value of tulathromycin was measured as described previously (30).

The MPC assay was performed as previously described with some modifications (31). Briefly, 1 L logarithmic phase *M. hyopneumoniae* cultures were centrifuged at 3,000 rpm for 10 min and the supernatant was removed and the pellet was reconstituted with fresh media to obtain ≥10^9^ CFU/mL and 200 μL was spread-plated on tulathromycin agar plates and incubated as per above for 7 d. The lowest drug concentration contained in the plate without colony formation was determined as the primary MPC (MPCpr). Using MPCpr as the baseline, the concentration of 10% drug was linearly decreased to 0.5 MPCpr and the previous steps were repeated. The final drug concentration was taken as the MPC. Each experiment was repeated three times.

### Time–Kill Curves

These experiments utilized 3.5 mL blank medium, 0.1 mL tulathromycin dilution with final concentrations at 0, 1/2, 1, 2, 4, 8, 16 and 32 × MIC and 0.4 mL logarithmic *M. hyopneumoniae* that were added to a 10 mL bottle. The amount of *M. hyopneumoniae* to give a final CFU/mL of 10^6^ followed by standard incubation conditions for 48 h as per above. Samples of 100 μL were taken at 0, 3, 6, 9, 12, 24, 36 and 48 h and counted. The CFU were determined via 10-fold serial dilutions and plating 10 μL of each diluted sample on drug-free agar. The growth control group (no antibiotics) and the blank control group (no *M. hyopneumoniae*) were included in the experiment. The results were read a week later to plot the kill curves. The detection limit was 100 CFU/mL and the test was repeated three times.

### PK/PD Dynamic *in vitro* Model and Dosing Regimens

The one-compartment model with first order absorption could theoretically describe the intramuscular injection process better, but it was not suitable for this study. We fitted the lung pharmacokinetic data given in the literature (32) and calculated K_a_ and K_el_ to be 0.178 and 0.0048; the t_1/2Ka_ and t_1/2Kel_ were 3.9 h, 144 h. *M.hyopneumoniae* grew slowly, and in long experiments, the absence of a brief absorption period had little effect on the results. Meanwhile, the short t_1/2Ka_ resulted in a small volume of the absorption bottle, so the one-compartment model with first order absorption will affect the accuracy of the experiment.

We constructed an *in vitro* model to simulate an intravenous injection one-compartmental model (33–35) and consisted of three parts: (1) the reserve bottle providing fresh medium (2) the reaction bottle simulating drug target organ containing 300 mL medium and 10^8^ CFU/mL *M. hyopneumoniae* in a 10 mL semi-permeable membrane and (3) the excretion bottle. The three sections are connected by plastic hoses and a peristaltic pump provides a steady flow rate to the unit. The elimination process of tulathromycin in this device simulates the first-order rate:


$$\begin{array}{l} C=C_{0}\times e^{-kt} \end{array}$$

where *C*_0_ was the initial concentration of the drug, *C* was the concentration of the tulathromycin at *t, k* was the elimination rate constant and t the post-administration time. The elimination half-life of tulathromycin in pigs has been previously determined to be 144 h, and the clinically recommended dose is 2.5 mg/kg (32). The peristaltic pump flow rate was set according to the elimination half-life of drugs to simulate the elimination process of drugs *in vivo*. In brief, tulathromycin was injected into the reaction bottle and the semi-permeable membrane at the same time so that the drug concentration inside and outside the membrane quickly reached equilibrium. The initial concentrations of tulathromycin were 0, 1, 2, 4, 8, 16, 32, and 64 × MIC. At 6, 9, 12, 24, 48, 72, 96, 120, 144, and 168 h after administration, 2 mL samples were collected to determine the drug concentration. All samples were stored at −20°C.

### Determination of Tulathromycin Concentrations in Media

The sample processing and detection methods for tulathromycin detection were modified as outlined previously (32). In brief, 1% acetic acid in acetonitrile was used for drug extraction prior to HPLC-MS/MS analysis using a Luna C18 (150 mm × 2 mm, 5 μm) column with mobile phase A (water with 0.1 % formic acid) and B (acetonitrile) using the following gradient: 0–3 min: 10 to 90 % B and maintain from min 3 to 5 min at 90 % B. 5–5.5 min: 90–10% B and maintain from 5.5 to 8 min at 10% B. Flow rate: 250 μL/min; column temperature: 40°C and injection volume 5 μL.

### *In vitro* Dynamic Time-Kill Curve Fitting and Sensitivity Analysis

The model as indicated above was used and 0.3 mL samples were taken from the reaction flask every 12 h until 168 h and CFU were determined by plating on drug-free plates and plates containing tulathromycin at 1 × MIC. Ten colonies selected at 12, 36, 60, 84, 108, 132 and 168 h were diluted 1,000 × and cultured as per standard conditions (see above) until the color changed from rose red to orange. The cultures were used to extract DNA for amplification and sequencing. Single colonies from the drug plate at 168 h were inoculated into fresh medium and the MIC was measured when they reached logarithmic growth phase and samples were stored −80°C. PK/PD parameters were fitted using WinNonlin 5.2.1 (Certara, Princeton, NJ, USA). The PK/PD parameters included the cumulative time at which the concentration exceeded the MIC (%T> MIC), the peak concentration divided by MIC(Cmax/MIC) and the area under the concentration-time curve over 72 h divided by the MIC (AUC_72h_/MIC). This test was assessed using the Sigmoid E_max_ inhibition model as follows:


$$\begin{array}{l} E=E_{\max}-\frac{\left(E_{\max}-E_{0}\right)\times C_{e}^{N}}{EC_{50}^{N}+C_{e}^{N}} \end{array}$$

Where the following are represented: *E*, Antibacterial effect; *E*_max_, variation for the bacteria in the control group after 72 h (ΔLog_10_ CFU 72 h / mL); *E*_0_, Maximum antibacterial effect after 72 h; EC_50_, Ce when reaching 50% of the maximum antibacterial effect; Ce, PK/PD model parameters (including *C*_max_/MIC, %T> MIC, AUC_72h_/MIC). In this study, Ce is AUC_72h_/MIC; *N*, slope of the dose-response curve.

For the enrichment of drug-resistant bacteria, a simple *E*_max_ model was used to describe the growth process of drug-resistant bacteria, and the equation is as follows:


$$\begin{array}{l} E=\frac{E\max\;\times C_{e}^{N}}{EC_{50}^{N}+C_{e}^{N}} \end{array}$$

*E*_max_, The maximum concentration of drug-resistant bacteria can be achieved by the model. *E*_0_, In the absence of antibiotics, the production of resistant mutants. EC_50_, PK/PD model parameter value at half of maximum effect. Ce, see above. In this study, Ce is AUC_168h_/MIC.

### DNA Extraction and Sequencing

DNA was extracted using a Mycoplasma gDNA Miniprep Kit as previously described (36). The genes encoding the 23S rRNA domain V, L4 and L22 regions were amplified and sequenced (36). The sequencing results were compared with *Escherichia coli* and *M. hyopneumoniae* ATCC 25934 using DNASTAR (Madison, WI, USA) to identify mutations.

### Analysis of Efflux Pump Mechanism

In order to detect whether efflux pump mechanism exists in drug-resistant strains, we re-measured MICs of macrolides and tiamulin against all drug-resistant strains in the presence of reserpine. In the presence of reserpine, MIC values of strains with efflux pump mechanism decreased.

Efflux pump inhibition assessed susceptibility to tulathromycin, tiamulin, tilmicosin and tilmicosin in the presence of 5 μg / mL reserpine. Previous studies have reported that 20 ug/mL reserpine can inhibit the active effusion of *M. hominis* to drugs (18), but this concentration has a significant effect on the growth of *M. hyopneumoniae*. The optimal reserpine concentration was determined using an *M. hyopneumoniae* culture at 10^6^ CFU/mL containing reserpine (final concentration at 0, 5, 10 and 20 μg/mL) that were added to 96-well plates and observed after 48 h for the culture color change (see above). A final reserpine concentration of 5 μg/mL resulted in a simultaneous color change consistent with the growth control (reserpine 0μg/mL). To further prove that 5 μg/mL reserpine had no effect on the growth of *M. hyopneumoniae*, we measured the growth curve in the presence of reserpine. Growth curves carried out in the presence of reserpine at 0, 5 and 20 μg/mL were measured. In brief, exponential phase cultures were inoculated into fresh medium to obtain an initial level of 10^6^ CFU/mL and allowed to grow for 96 h, Samples were taken every 24 h and CFU were determined on drug-free agar plates.

### Real-Time PCR

Real time PCR was conducted using primers specific for the *M. hyopneumoniae* efflux pump genes and the 23S rRNA gene that was used as an internal standard (Supplementary Table). Gene sequence reference Ana Tereza R Vasconcelos et al. (37). The strains were inoculated in drug-free medium and cultured to exponential phase for total RNA extraction. RNA samples were used to prepare cDNA. The analysis was conducted as previously reported (38). In brief, RNA was extracted using the TransGen RNA Kit (TransGen Biotech, Beijing, China) and cDNA was prepared using the Goldenstar RT6 cDNA Synthesis Kit (Tsingke, Beijing, China). The reaction mixtures contained 0.8 μL each primer (10 μmol/L), 10 μL of 2 × SYBR buffer and 2 μl cDNA in 20 *u*L total volume. The thermal cycling conditions were as previously reported (38). All assays were repeated three times, and the data were analyzed by 2^−ΔΔCT^ method to calculate the expression of each gene (39). At the same time, the gradient dilution of the mRNA sample was used for RT-PCR, and the regression curve of sample concentration-*C*_*T*_ value was plotted. The following equation was used to calculate the amplification efficiency (*E*) of candidate gene primers (40). *R*^2^ was used to estimate the reliability of regression equation of standard curve.


$${\text{E }=\;(10}^{-1/\text{slope}}-1)\;\;\times \;\;100$$

The software IBM SPSS was used to conduct an one-sample *T-*test for the experimental results, and the *P*-value reflected whether the expression differences were significant.

### Analysis of *in vitro* Adaptability

Growth curves for the co-cultivation and individual cultivation of mutants and the parental strain was carried out using incubation conditions as per above. In brief, exponential phase cultures were inoculated into fresh medium to obtain an initial level of 10^3^ CFU/mL and allowed to grow for 168 h. Samples were taken at regular intervals and CFU were determined on drug-free agar plates. During co-cultivation, equal volumes of mutant and parent were added to fresh medium. Plain agar plates and drug plates containing 1 × MIC drug levels were used for CFU counting. OriginPro software was used to calculate the growth kinetic parameters for each strain. Competition experiments were used to calculate adaptability as previously described with some modifications (41). In brief, the initial co-cultivation level for inoculation was 10^4^ CFU/mL and CFU counting was performed after 96 h. The relative fitness (*W*) of mutants and wild type were calculated as follows:


$$\begin{array}{l} \text{W}=\frac{{\text{Log}}_{\text{10}}(\text{RF}/\text{RI})}{{\text{Log}}_{\text{10}}(\text{SF}/\text{SI})} \end{array}$$

Where *W* is the relative fitness value. RF, the amount of drug-resistant bacteria at the end of culture; RI, the amount of drug-resistant bacteria before culture; SF, the amount of sensitive bacteria at the end of culture; SI, the amount of sensitive bacteria before culture.

## Results

### Susceptibility Determinations

The MICs of the parental *M. hyopneumoniae* strain ATCC 25934 to tiamulin, tulathromycin, tylosin and tilmicosin were determined to generate a baseline for succeeding experiments. The standard micro-dilution method generated MIC values of 0.08, 0.3125, 0.0625 and 1.6 μg/mL for these 4 drugs, respectively. The MICs for tiamulin, tulathromycin and tilmicosin using the agar dilution method were 0.32, 0.625 and 12.8 μg/mL. As the two methods have different criteria for results, MIC results are different. The standard micro-dilution method was used to judge MIC results by color changes, and agar dilution method was used to judge MIC values by colony growth. A small amount of living organisms is not enough to change the color of the medium, which is responsible for the MIC differences. In addition, the MPC for tulathromycin was 80 μg/mL and the MSW range was 0.3125–80 μg/mL. We re-measured MICs of macrolides and tiamulin against all drug-resistant strains in the presence of reserpine. Only MIC values of MTIL1, MTIL2 and MTIL3 were different, and the results were marked with ^*^ (Table 1).

**Table 1:** MIC and MPC of four antibiotics against *M. hyopneumoniae* and mutants.

**Strains**	**Micro-dilution MIC (μg/mL)**				**Agar-dilution MIC (μg/mL)**			**Mutation (***E.coli*** numbering)**
	**Tulathromycin**	**Tiamulin**	**Tilmicosin**	**Tylosin**	**Tulathromycin**	**Tiamulin**	**Tilmicosin**	
ATCC25934	0.3125	0.08	1.6	0.0625	0.625	0.32	12.8	-
MTUL1	320	0.16	1638.4	64	>320	0.64	>320	A2058T
MTUL2	320	0.16	1638.4	32	>320	0.64	>320	A2058G
MTIA1	0.3125	0.32	1.6	1	0.625	2.56	12.8	A2059T
MTIA2	0.3125	0.64	1.6	1	0.625	2.56	12.8	A2059T
MTIL1	160/0.3125*	0.08/0.08*	819.2/3.2*	8/0.125*	320	0.32	>320	–
MTIL2	160/0.3125*	0.08/0.08*	1638.4/6.4*	16/0.125*	320	0.32	>320	–
MTIL3	320/80*	0.16/0.08*	1638.4/409.6*	16/8*	>320	0.64	>320	A2058G

* *MIC in the presence of 5 μg/mL reserpine*.

### Analyses of Time–Kill Curves

**Figure 1:**
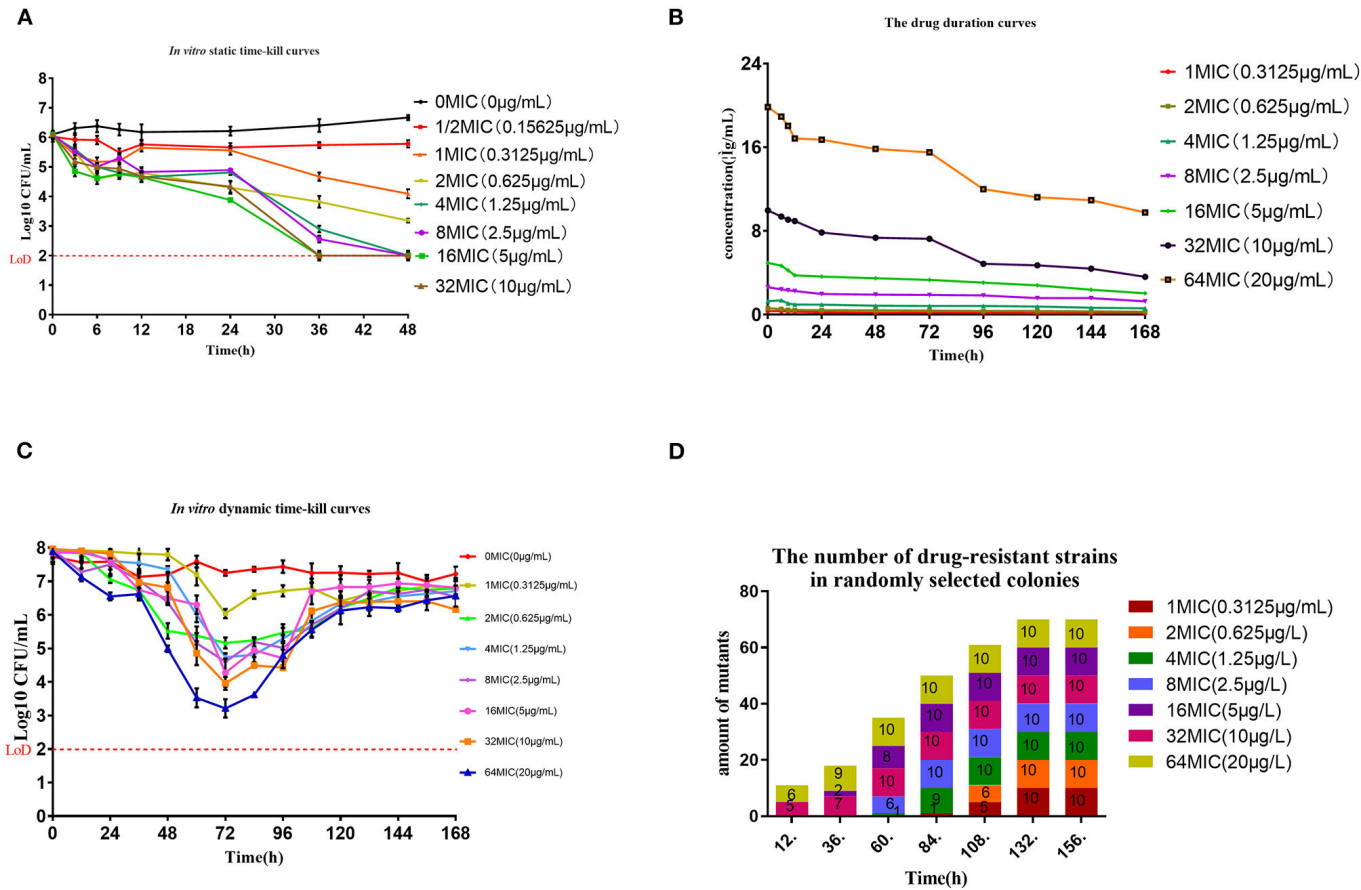
Pharmacokinetic and pharmacodynamic results in dynamic models. **(A)** Time-kill curves for tulathromycin at constant concentrations. The red dash represents the method detection limit **(B)** Concentration-time curves for 7 levels of tulathromycin for the in vitro dynamic model. **(C)** Dynamic time-kill curves depicted at 0–64 × MIC for tulathromycin. The initial concentration of tulathromycin in the reaction bottle. The red dash represents the method detection limit **(D)**. Numbers of 23S rRNA mutants in colonies collected from the reaction bottle every 24 h.

*In vitro* static time-kill curve indicated that after treatment at 0 – 32 × MIC tulathromycin for 48 h, the reduced bacterial populations were from 0.24 to 4.1 Log_10_ CFU/mL. At 0.5 – 2 × MIC, tulathromycin had an inhibitory effect on *M. hyopneumoniae* and the reduced bacterial population were from 0.24 to 2.88 Log_10_ CFU/mL. When the drug concentration was ≥ 4 × MIC, a bactericidal effect was observed within 48 h and the maximum bacterial population had decreased by 4.1 Log_10_ CFU/ mL, At 16 – 2 × MIC. The population of bacteria decreased to the detection limit at 36 h, maybe the population of bacteria was lower. The detection limit was 100 CFU/mL (2 Log_10_ CFU/mL) (Figure 1A).

### *In vitro* Dynamic Model and Effects of Changing Tulathromycin Concentrations on Sensitivity of *M. hyopneumoniae*

The drug duration curves (Figure 1B) for tulathromycin were all within the mutant selection window (MSW) of 0.3125–80 μg/mL except for 1 × MIC. An HPLC method was developed to detect tulathromycin concentrations and we found limits of detection (LOD) and quantitation (LOQ) at 1 and 5 ng/mL, respectively, and recoveries of 74.13 ± 3.29% (*n* = 15). The intra-assay coefficients of variation (CV) were 4.29, 6.75 and 5.14% (*n* = 5) while the inter-assay CVs were 5.43, 6.21 and 6.49% (*n* = 15). The *in vitro* dynamic sterilization curve indicated that the decrease in bacterial numbers for each dose group was the largest at 72 h where the maximum antibacterial effect was achieved. A level of 4 × MIC could achieve a bactericidal effect where bacterial numbers decreased by 3.19 Log_10_ CFU /mL, and the growth of each dose group resumed after 72 h (Figure 1C). The AUC_72h_/MIC had the highest correlation among the three parameters (*R*^2^ = 0.987) and the antibacterial effect of tulathromycin on *M. hyopneumoniae* was concentration-dependent (Table 2).

**Table 2:** Estimation of PK/PD parameters, and data are derived from the *E*_*max*_ model.

**PK/PD parameter**	***E*** **_max_ (Log_10_ CFU/mL)**	**EC_**50**_**	***E*** **_0_ (Log_10_ CFU/mL)**	**Slope (***N***)**	***R*** **^2^**
AUC_0−72_/MIC (h)	−0.47	1303.91	−6.05	0.38	0.9929
*C*_max_/MIC	−0.47	8.21	−6.32	0.41	0.9867
%T>MIC	−0.46	17.45	−4.12	0.80	0.8964

**Figure 2:**
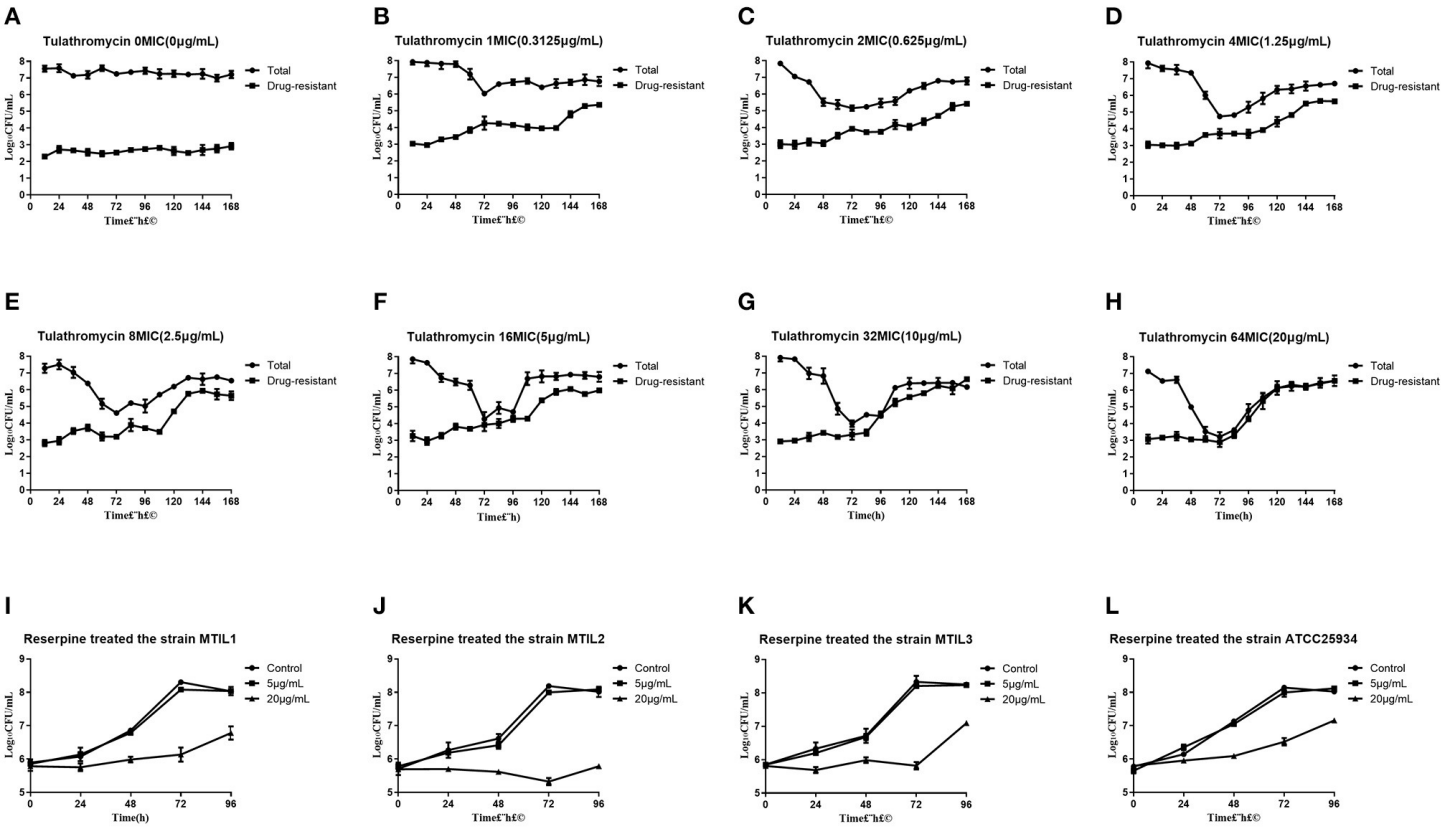
**(A–H)** Total and resistant bacterial CFU at the indicated initial concentration in the reaction bottles. **(I–L)** Growth conditions of 4 representative strains in the presence of reserpine as indicated. To verify whether reserpine affects strain growth. Error bars represent standard deviation.

In general, we found that bacteria numbers at the plateau stage of growth increased according to the administration dose. The region between the two curves represented sensitive bacteria that had not been killed. In the high-dose groups of 32 and 64 × MIC, the total numbers were coincident with the drug-resistant bacterial curve after 72 h. This indicated that all the sensitive bacteria had been replaced with drug-resistant bacteria (Figures 2A–H). By estimating the AUC and the increase of drug-resistant bacteria through the *E*_max_ model, we calculated that the *E*_max_ was 2.93Log10 CFU/mL, *E*_0_ was 0.27Log10 CFU/mL and the EC_50_ was 111.87 (Table 3). The maximum enrichment amount of drug-resistant bacteria in the whole model predicted by the software is 2.93, and when the AUC/MIC is only 111.87, it can reach half of *E*_max_, indicating that low dose drugs can achieve good enrichment effect. In the absence of drugs, the model spontaneously produced drug-resistant bacteria due to the mutation of individual bacteria cultured for a long time. Samples were collected for sequencing every 24 h. and the proportion of 23S rRNA mutants increased according to dose and mutants appeared earlier and accounted for a higher proportion with increasing dose. All samples tested after 108 h were 23S rRNA mutants (Figure 1D).

**Table 3:** Estimation of AUC_0−168_/MIC (h) and increases in antibiotic-resistant bacteria.

**Parameter**	**Value**
*E*_max_ (Log_10_ CFU/mL)	2.93
EC_50_	111.87
*E*_0_ (Log_10_ CFU/mL)	0.27
*R* ^2^	0.9761

### Resistance Analysis

The numbering of mutation sites is based on *Escherichia coli*. All the drug-resistant strains we found in our screens from the *in vitro* model for tulathromycin were 23S rRNA A2058G mutations except for strain MTUL1 that was A2058T. MTUL1 and MTUL2 were selected for further experiments. In contrast, we found no mutations for the tilmicosin-resistant strains MTIL1 and MTIL2 while MTIL3 possessed an A2058G mutation. The binding site of pleuromutilins to ribosomal RNA is different from that of macrolides. In order to explore the cross-resistance of two types of resistant strains, we conducted a study. For tiamulin resistance, A2059T was found and was represented by the mutants MTIA1 and MTIA2. The mutants screened from macrolides showed significantly increased resistance to macrolides, and their MICs increased by many times, but only slightly increased MIC values to tiamulin. At the same time, MTIA1, MTIA2, was only slightly less sensitive to the antibiotics used in this study. There was cross-resistance among macrolide-resistant strains, and there was no cross-resistance between macrolide-resistant mutants and pleuromutilin-resistant mutants (Table 1).

### Analyses of Efflux Pump Mechanism

**Figure 3:**
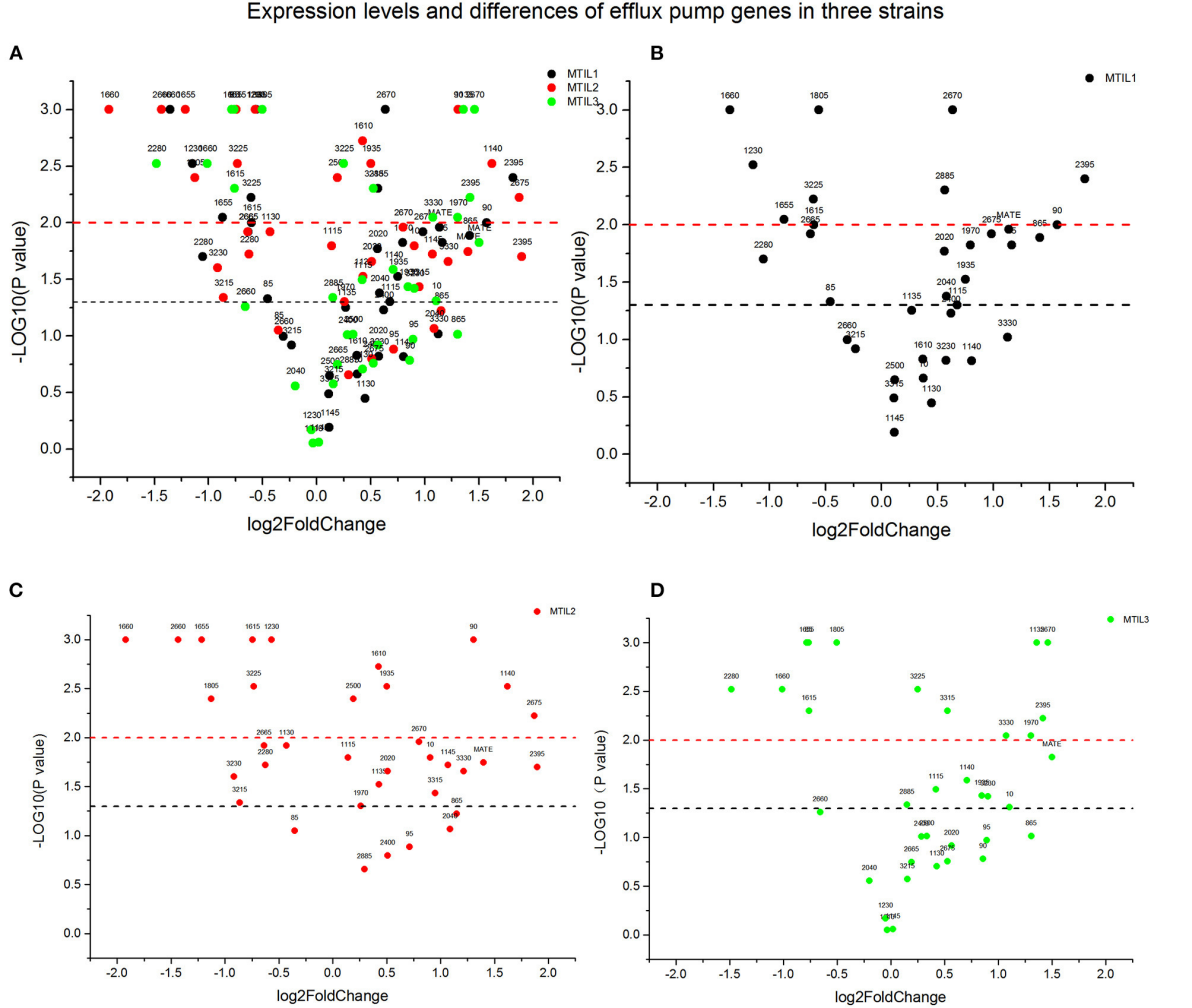
Expression of genes associated with active efflux in the indicated strains. The *Y*-axis number represents the negative logarithm of the *p*-value (The black dash represents *P*-value = 0.05, significant difference; The red dash represents *P*-value = 0.01, extremely significant difference). The values of the X-axis are expression quantity. Strains MTIL1, MTIL2 and MTIL3 are represented by the black, red and green spots, respectively. The numbers above the spots represent the gene numbers (see Supplementary Table). **(A)** Comparison of gene expression levels of efflux pump in three strains. **(B–D)** The efflux pump genes expression levels of the three strains were respectively displayed.

We found that 5 μg/mL reserpine had no effect on the growth of ATCC 25934 and the drug-resistant strains while 20 μg/mL was inhibitory (Figures 2I–L). We tested the efflux pump mechanism in all resistant strains with efflux pump inhibitor reserpine. In the presence of reserpine, only the MICs for the tilmicosin-resistant strains decreased. The MICs for tilmicosin, tulathromycin and tylosin against MTIL1 and MTIL2 decreased by 256, 512 and 64 ×. MTIL3 still had a high level of resistance to these macrolides and the MIC decreased by 4, 4 and 2×, respectively (Table 1). For MTIL1 and MTIL2, they were highly susceptible to macrolides after adding reserpine, and their MIC values were consistent with standard strain ATCC25934. The development of drug resistance in these 2 strains was mostly caused by efflux pump activity. In the presence of reserpine, The MIC value of MTIL3 decreased, but resistance remained. It has A2058 mutation in the domain V of 23S RNA. We therefore screened for the expression of 35 ABC transporter genes and MATE efflux transporter, the amplification efficiency was >90% with correlation coefficient >99%. We investigated the expression differences of ABC and MATE transporters in MTIL1-3 in drug-free medium compared with sensitive strain. Expression of RS01935, RS02670, RS01115, RS01970, RS02395 and MATE family efflux transporter (RS03540) were up-regulated (*P* < 0.05 or *P* < 0.01) in all 3 strains. There were also 5 significantly down-regulated genes including RS01615, RS01655, RS01805, RS01660 and RS02280 (*P* < 0.05 or *P* < 0.01). The other genes were differentially expressed. The same gene between different strains was also expressed differently in the corresponding sensitive strains in a case-by-case basis (*P* > 0.05) (Figure 3).

### *In vitro* Growth and Competition

**Figure 4:**
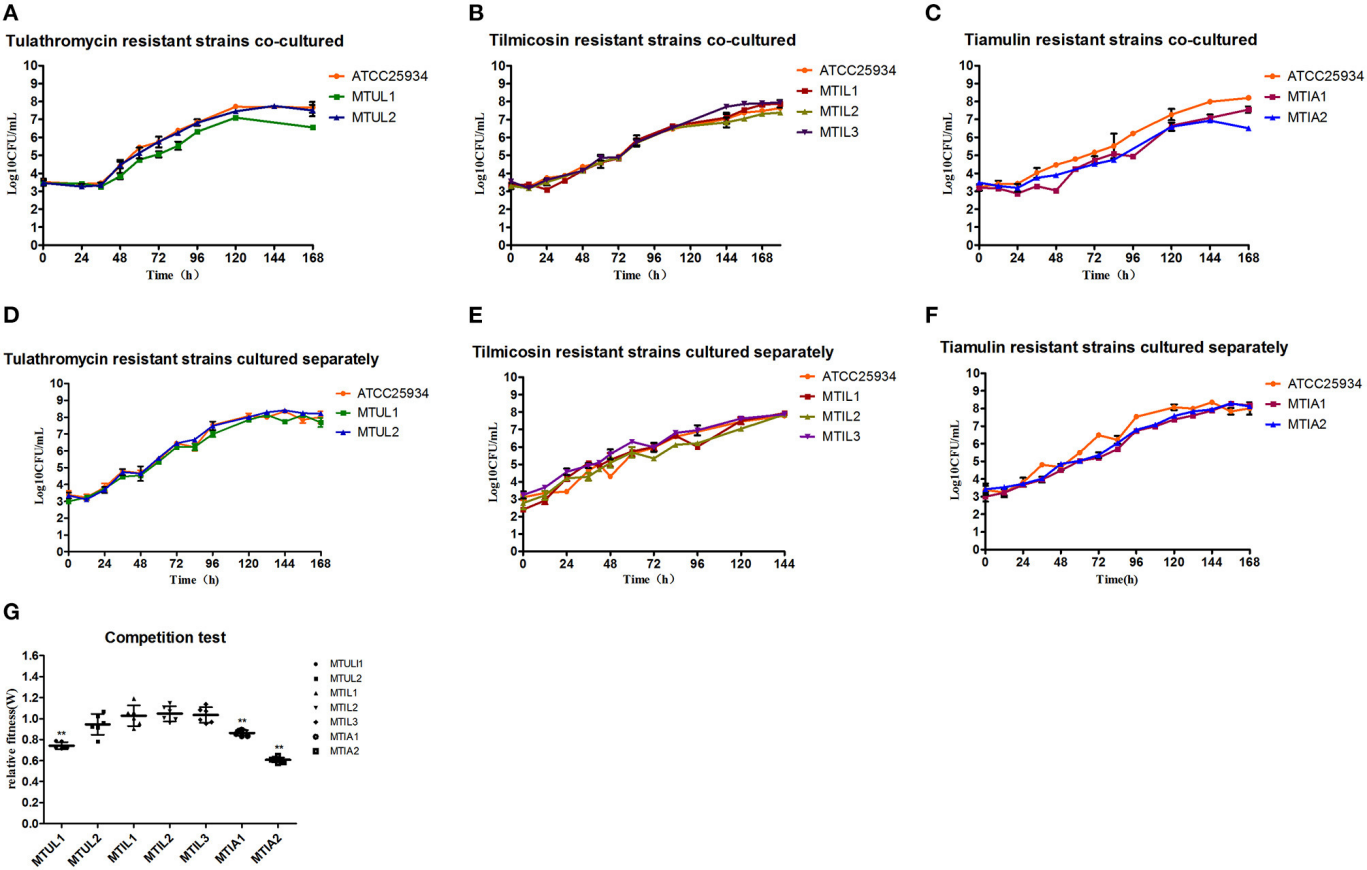
Fitness cost analysis of 23S rRNA mutants. **(A–C)** Growth curve of drug-resistant mutants and parental strains in co-culture. **(D–F)** Growth of drug-resistant mutants and parental strain cultured separately. **(G)** Relative fitness of all 7 mutants. Error bars show standard deviations. **P < 0.01.

The drug-resistant mutants when cultured separately displayed overlapping growth curves with no significant differences between the growth rate and quantity at the stationary stage. When the parental strain ATCC 25934 and mutants were co-cultured, the MTUL1, MTIL2, MTIA1 and MTIA2 curves were below that of ATCC 25934. These differences were 1.12, 0.25, 0.68 and 1.69 Log _10_ CFU/mL, respectively. In contrast, the curve for MTIL3 was higher than the parental strain while MMTUL2 and MTIL1 curves were almost coincident (Figure 4). In co-cultured, MTUL1 had a longer generation time (G) than ATCC 25934 and MTUL2. However, this parameter of the three strains were not significantly different when cultured separately. There was no significant difference in generation time among the three tilmicosin-resistant strains cultured alone or with sensitive strains. However, the generation time of tiamulin-resistant strains was lower than that of sensitive strains in both cultures (Table 4).

**Table 4:** Growth kinetic parameters of drug resistant strains and parental ATCC 25934.

**Strains**	**ATCC25934a**	**MTUL1**	**MTUL2**	**ATCC25934b**	**MTIL1**	**MTIL2**	**MTIL3**	**ATCC25934c**	**MTIA1**	**MTIA2**
*G*(h)	5.81/6.83	5.94/8.51	5.65/6.97	6.65/ 9.55	5.08/9.60	7.31/9.68	5.84/9.49	5.82/8.45	7.85/9.22	8.07/11.92

*Growth kinetic parameters values are expressed as “single-cultured/co-cultured”*.

The competition experiments also indicated that the relative fitness (W) for MTUL1, MTIA1 and MTIA2 were significantly lower than that of the antibiotic sensitive strains (*P* < 0.01) and were 0.74, 0.86, and 0.61, respectively. There were no significant differences in W values for MTUL2, MTIL1, MTIL2, MTIL3 or for the antibiotic sensitive strains (*P* > 0.05) (Figure 4). These data indicated that the A2058T and A2059T mutants might be gradually replaced by sensitive strains under co-culture and the strains containing efflux pump and A2058G mutants were comparable in competitiveness with the sensitive strains.

## Discussion

*M. hyopneumoniae* is the primary pathogen causing respiratory diseases in intensive pig farming around the world. This organism is very difficult to isolate and culture *in vitro* and its growth cycle is long. Therefore, there have been few reports on the PK/PD synchronization model of antibiotics used against *M. hyopneumoniae* or the fitness costs. We used an *in vitro* peristaltic pump model to evaluate the PK/PD relationships between tulathromycin and *M. hyopneumoniae* and the emergence of resistant bacteria. The purpose was to determine whether the effects of tulathromycin are concentration- or time-dependent for this pathogen and to predict the best PK/PD parameters for the curative effect of tulathromycin. The drug resistance mechanism of *M. hyopneumoniae* was explored and the fitness cost of resistant mutant strains was reported. This data will assist in assessing transmission risks of drug-resistant strains.

The activity of tulathromycin against *Haemophilus parasuis, Pasteurella multocida* and *Streptococcus suis* is bactericidal within 12 h (42–44). We found that after 24 h of drug exposure for *M. hyopneumoniae*, the overall growth effects were inhibitory and after 48 h the action was bactericidal. Our calculations indicated that the action time of tulathromycin was longer than previously reported and is the reason that we used the longer sampling time in our studies. There are no reports for the PK/PD of tulathromycin against *M. hyopneumoniae*.

Tulathromycin is a macrolide and is primarily used as a bacteriostatic agent. Antibacterial drugs are classified into time-dependent (β-lactams) or concentration-dependent (aminoglycosides). For macrolide drugs, most data indicated that the antibacterial activity of these drugs was related to %T>MIC (45), but there were also studies reporting that the half-life of azithromycin in the body was so long that its antibacterial activity was related to AUC_24h_/MIC (46). Inhibitors can also show bactericidal activity at higher concentrations (47, 48). The antimicrobial activity of antibiotics against bacteria has been determined using treatment time and concentration. The *in vitro* dynamic model that we applied indicated that the antibacterial effect of tulathromycin on *M. hyopneumoniae* was positively correlated with the administered dose and similar to the results of the static sterilization curves. The bacterial counts were at their lowest at 72 h so the bacterial counts from 0 to 72 h were used for PK/PD fitting. The AUC72h/MIC had the highest correlation with antibacterial effect (*R*^2^ = 0.993), Cmax/MIC had the second highest correlation with antibacterial activity and %T>MIC had a low correlation with antibacterial effect (*R*^2^ = 0.896) (Table 3). These data indicated that the antibacterial effect of tulathromycin on *M. hyopneumoniae* was concentration-dependent.

Tulathromycin accumulates in lung tissues with a long half-life and the MIC of tulathromycin to *M. hyopneumoniae* was low. The drug concentration could be maintained above the MIC for a long time after one administration and this was the reason why %T> MIC cannot guide medication dosing. After 72 h, we found that the bacteria in each dose group resumed growth and the bacterial population was about 7 Log _10_ CFU/mL at hour 168. The long half-life also resulted in the drug concentration in the reaction bottle was 1/2 C0 after 168 h of culture and high bacterial loads altered tulathromycin into a growth promoter of drug-resistant bacteria. The EC50 was 111.87 and proved that low concentrations of the drug can still enrich drug-resistant bacteria even after extended periods of exposure. From the drug time curve, most drug concentrations fell within the MSW and lasted for a long time and this provided the basis for the targeted selection of drug-resistant mutants. For example, a previous study demonstrated that the longer linezolid was maintained at the MSW, the easier it was for enrichment of linezolid-resistant *Staphylococcus aureus*. The enrichment of drug-resistant bacteria was positively correlated with time inside the MSW (T_MSW_) (49). Additionally, when danofloxacin levels for *Mycoplasma* were at the lower and middle parts of the MSW using an *in vitro* dynamic model, *Mycoplasma gallisepticum* was prone to drug resistance. When the drug concentration was outside the MSW, no change in bacterial susceptibility was found (50). The region between our curves representing total bacterial counts and the drug-resistant bacteria in the middle-dose group most likely represent the persistent strains while the drug-resistant bacteria in the high-dose group gradually replaced sensitive bacteria. The persister is a special bacterial form that allows the bacteria to cope with adverse environments. Bacteria can tolerate the antibiotic environment by lowering metabolism for self-protection, but drug sensitivity does not change after reactivation (51–53). Our sequencing results indicated that the mutants appeared earlier than the results of bacterial counts indicated. The reason was most likely that the growth of strains without drug-resistant mutations was affected and the colony was too small to select. This led to the earlier emergence of mutants.

*M. hyopneumoniae* colonies are so small that they cannot be observed with the naked eye so picking single colonies is very difficult. In *Brucella* a single nucleotide polymorphism (SNP) method was developed using RT-PCR to resolve similar problems (54). However, RT-PCR needs to determine the location and form of mutation and cannot be used to detect unknown mutations. Our *in vitro* model results indicated that tulathromycin had a bactericidal effect on *M. hyopneumoniae* and drug-resistant bacteria were easily enriched and resumed growth. This indicated that in the process of clinical medication, the single use of tulathromycin therapy has the risk of inducing resistance. The use of doxycycline and azithromycin in succession for *Mycoplasma genitalium* therapy indicated a cure rate of 95.4% and resistance levels of only 4.6% (55). Doxycycline combined with sitafloxacin also had a good therapeutic effect on multidrug-resistant *M. genitalium*. At the same time, under this combined treatment there was only one *par*C G248T / S83I mutation and no *gyr*A mutations (56). Therefore, rotation and combination of medications provide a way to prevent drug resistance.

Macrolides and pleuromutilins bind to the 50S ribosomal so in the current work, we sequenced the 23S rDNA L4 and L22 regions. Interestingly we did not find mutations in L4 and L22 similar to the results of previous studies (20). The tilmicosin-resistant strain MTIL3 and tulathromycin-resistant strain MTUL2 were the result of A2058G mutations and are common in macrolide resistance in *M. genitalium* (57), *Mycoplasma bovis* (58), *Mycoplasma capricolum* (59), *M. gallisepticum* (60), *M. hyopneumoniae* (19) and *M. hominis* (10). In the dynamic model, most of the drug-resistant bacteria isolated were A2058G mutants and only one strain possessed A2058T (MTUL1). The latter was rare in *M. hyopneumoniae* and has since been reported in *M. genitalium* (61). The A2058G and A2058T mutations caused high-level resistance of *M. hyopneumoniae* to all 3 of our test macrolides, and slightly increased the MIC of tiamulin. The 23S rRNA V region that contained the 2,058 and 2,059 mutations cause a variety of bacteria to high-level resistance to macrolides, but the A2059T mutant in our experiments was only slightly resistant to tiamulin and tylosin. In a similar manner, the single occurrence of A2059T *Neisseria* spp. resulted in a sightly increase in azithromycin resistance (62). The strains studied in our experiments contained only single mutations and the MIC of tiamulin only slightly increased. The 2,058 and 2,059 positions of 23S rRNA and tiamulin molecules do not directly interact (23) and is consistent with our results for tiamulin sensitivity. So mutations in these two sites do not lead to high levels of tiamulin-resistance. There was no cross-resistance between macrolide—resistant strains and tiamulin-resistant strains. The resistance profiles of the two types of drug-resistant strains were different, which was related to the binding site of drug molecules at 23srRNA. The simultaneous mutations of multiple sites bring about strong tiamulin resistance such as reported for *M. gallisepticum* where the A2503U mutation when combined with other mutations increased the MIC to this drug (63).

The macrolide sensitivity of MTIL1, MTIL2 and MTIL3 elevated in our efflux pump experiments with the addition of reserpine. A comparative genomic analysis had indicated that the efflux pumps of *M. hyopneumoniae* were primarily ABC and MATE family transporters (12). After the addition of reserpine, MIC levels of MTIL1 and MTIL2 were consistent with those of sensitive strains, so we highly suspected that the drug resistance of these two strains was mediated by the efflux pump. Because their 23sRNA sequence did not mutate, their MIC values were consistent with those of sensitive strains when the efflux pump was inhibited. After the addition of reserpine, THE MIC of MTIL3 decreased fourfold, but it remained resistant to macrolides. Meanwhile, this strain is a mutant of A2058, and the mutation of 23SRNA contributes a part of macrolides resistance. To test this hypothesis, we investigated the expression of efflux pump genes. The existence of MATE family efflux transporters in *M. hyopneumoniae* has been clearly reported, but which ABC family transporters of *M. hyopneumoniae* are associated with active efflux of antibiotics has not been clearly proposed. ABC transporters are a class of permeable enzymes responsible for the transport of various substances in cells and play an important role in various physiological activities. We hoped to find ABC transporters associated with active efflux of drugs by RT-PCR. We found that the expression of MHJ_RS01935, MHJ_RS02670, MHJ_RS01115, MHJ_RS01970, MHJ_RS02395, and MATE family efflux transporter (MHJ_RS03540) were all up-regulated in the three strains (*P* < 0.05 or *P* < 0.01). The expression of other ABC protein genes was not fixed so *M. hyopneumoniae* could adjust the expression of different ABC proteins to reduce drug sensitivity (Figure 3). In addition, the expression of the MATE family efflux transporter (RS3540) in three strains were all up-regulated. These data indicated that ABC and MATE efflux pumps are actively used by *M. hyopneumoniae* in response to macrolides. Those genes that are down-regulated may code for proteins involved in other physiological activities and are affected by drug resistance. In addition, we used 23S rRNA as a reference gene for our expression studies and found it to be stable in the three strains. An evaluation of reference genes for *Gluconacetobacter diazotrophicus* indicated that the 23S rRNA gene was an appropriate reference (64). We cultured the strains in an antibiotic-free environment so that we could rule out the influence of antibiotics on ribosomal RNA expression. Ribosomal RNA expression was similar in susceptible and resistant strains. *M. hyopneumoniae* is small and difficult to culture and the final culture yields at the end of exponential growth is low and therefore requires several passages for typical experimental procedures. This may affect the level of the 23S rRNA gene and could be a deficiency for this assay and this must be monitored.

In the current study we also found that different 23S rRNA mutations resulted in different degrees of fitness costs to the hosts. When A2058T (MTUL1) and A2059T (MTIA1, MTIA2) were co-cultured with sensitive strains, their growth was slower and their relative fitness was lower, while the fitness of A2058G (MTUL2, MTIL3) mutants were consistent with or even higher than that of sensitive strain. The A2059G mutation that also occurs in *Neisseria gonorrhoeae* also causes this condition (65) and explains the reason for only one A2058T mutant in the tulathromycin resistant strains. Two A2059T mutant strains that were tiamulin-resistant had different degrees of fitness costs. The reason for this is currently unknown although mutations at the same site also had different effects on different strains. Previous studies have indicated that the A2058G mutation has no effect on *Mycobacterium* spp. but causes an increase in the fitness cost of *Helicobacter pylori* in the absence of drug selection. In addition, conformational polymorphisms at sites 2,057–2,611 for the *Mycobacterium* 23S rRNA also affected the fitness cost of A2058G mutants (66). The G74D modification in *C. jejuni* L4 also increases the fitness cost of the G2073A mutation (67).

In conclusion, the treatment of swine enzootic pneumonia with tulathromycin for 72 h achieves a good antibacterial effect but drug-resistant bacteria can easily emerge. Single nucleotide mutation is difficult to cause cross-resistance between macrolides and pleuromutilins. The efflux pump mechanisms used by macrolide-resistant strains are related to the up-regulation of MATE family efflux transporter and ABC family transporter expression. When drug concentration changes dynamically, bacteria may develop drug resistance through multiple pathways, and there are different subgroups of drug resistant strains in a system. Additionally, the A2058T and A2059T 23S rRNA mutants were less competitive than the sensitive strains and no fitness cost was found for A2058G mutants. The growth of A2058G mutants in an antibiotic free environment can lead to the replacement of antibiotic sensitive bacteria and this must be a factor taken into consideration when formulating a treatment plan. The results showed that when the *C*_0_ was 1.25μg/mL (4 × MIC), the bactericidal effect could be achieved after 72 h, so the current clinically recommended dose of 2.5 mg/kg still had a therapeutic effect on *M. hyopneumoniae* infection. However, the bacteria recovered after 72 h, indicating that only increasing the drug dose could not solve the problem of bacterial resistance. The results of this study indicated that the wide MSW and long accumulation time of tulathromycin were the important inducement for bacteria to develop antibiotic resistance. The use of macrolides such as tulathromycin alone is not recommended in the treatment of chronic respiratory diseases caused by *M. hyopneumoniae*. From this perspective, the combination of two antibacterial agents to reduce the MSW range and change the drug when the bacterial load reaches the lowest are potential solutions.

## Data Availability Statement

The datasets presented in this study can be found in online repositories. The names of the repository/repositories and accession number(s) can be found in the article/Supplementary Material.

## Author Contributions

XX: conceptualization, investigation, formal analysis, data curation, and writing—original draft. LY and YL: investigation, data curation, and visualization. JY: formal analysis software and visualization. HD: methodology, resources, supervision, project administration, and funding acquisition. All authors contributed to the article and approved the submitted version.

## Funding

This work was supported by Local Innovative and Research Teams Project of Guangdong Pearl River Talents Program (2019BT02N054).

## Conflict of Interest

The authors declare that the research was conducted in the absence of any commercial or financial relationships that could be construed as a potential conflict of interest.

## Publisher's Note

All claims expressed in this article are solely those of the authors and do not necessarily represent those of their affiliated organizations, or those of the publisher, the editors and the reviewers. Any product that may be evaluated in this article, or claim that may be made by its manufacturer, is not guaranteed or endorsed by the publisher.
