# Supplementary material for: Emergence and Mechanism of Resistance of Tulathromycin Against Mycoplasma hyopneumoniae in a PK/PD Model and the Fitness Costs of 23S rRNA Mutants
Source: Front Vet Sci. 2022 Feb 11;9:801800. doi: 10.3389/fvets.2022.801800 (PMC8873822; doi:10.3389/fvets.2022.801800)
Supplement: Supplementary file 1 [file Table_1.docx]

Supplementary Table. PCR primers of efflux genes and reference gene used in this study

| Name | Primer |
| --- | --- |
| MHJ-RS01135 | F 5' GATGACTTTGGTTCTTCGGC 3'  R 5' CTTGCTCTTTTAATTTGGGC 3' |
| MHJ-RS01140 | F 5' TTTTCATTACTCACGACCTTGG 3'  R 5' TTCATCTCTTGTACCTTCCTCG 3' |
| MHJ-RS01145 | F 5' AAAAATATTTACCGCGCCCTT 3'  R 5' TTCCAGAACCAGATTCACCAAC 3' |
| MHJ-RS01230 | F 5' GAACTTGATACTGTAACCGCCG 3'  R 5' ATAATCGTTTTTCCAACCCCTC 3' |
| MHJ-RS01610 | F 5' AAGGTTACTTTCTCTTATGGCGA 3'  R 5' TTTTGATTGTTTGGTTCTTTTAT 3' |
| MHJ-RS01935 | F 5' TTATTTCGGCAACTGGGGT 3'  R 5' TGCCGGATGTCAACAGATT 3' |
| MHJ-RS02040 | F 5' CGGCTCAACCCTGCTTACA 3'  R 5' AAACAGTTGAAAGCCCAGC 3' |
| MHJ-RS02280 | F 5' TTCTTCCTTATAGTTTTCGGGC 3'  R 5' CAATTAATTGGTTTTGTGATCC 3' |
| MHJ-RS02500 | F 5' CGGCTTTTCCTCATTTAGT 3'  R 5' CTGATAGTGGCTTTTGATTTC 3' |
| MHJ-RS02665 | F 5' ATTCCTGAAGGGACTAACAAAGA 3'  R 5' CTAAAGCATATTCATTTCGTGGA 3' |
| MHJ-RS02670 | F 5' GTCCTTGTAAATGAGCAACTTCC 3'  R 5' ATCTGCCAGCGATTTGGG 3' |
| MHJ-RS02675 | F 5' TGGAACAGTTGCTGCTTATCGC 3'  R 5' TATTCAGAAGATAAAACGGTAA 3' |
| MHJ-RS02885 | F 5' TGGTTTTGAATGAACTACTCGC 3'  R 5' TGTAGCTGTATCACGTTTATGT 3' |
| MHJ-RS03220 | F 5' AGTCAATGGTAGGCAATCTTT 3'  R 5' TAAAAGATTGCCTACCATTGA 3' |
| MHJ-RS03225 | F 5' CTTTTCAGGAGTAATCGCAGGA 3'  R 5' AATGGCATCAAAACCTTCACG 3' |
| MHJ-RS03315 | F 5' TCAAGGAGTAAATGGTTCTGGA 3'  R 5' AGTTCCGTAAATAATACCTGGG 3' |
| MHJ-RS00085 | F:5'ATAAAAGCGAATGAAGAGAAAAACC 3'  R:5'TTGATTTAGTTTTTCACCTTGAGTATTA 3' |
| MHJ-RS00090 | F:5' CGACCTACAAACCCTCACTCAA 3'  R:5' TCATTTTCCGCTTAGAATCTGTTA 3' |
| MHJ-RS00095 | F:5' TCAAAGGTTGAGGCTGTTAGCA 3'  R:5'AAATCAACATACCCGTGACTACTTT 3' |
| MHJ-RS00100 | F:5' AAGTCAAAGATTTATCGGGTGG 3'  R:5' CAAGATTTCCGGTTGGTTCA 3' |
| MHJ-RS00865 | F:5' TGAACGTGTTGCCAAGGATT 3'  R:5' CATAATGATTTAGATCGCCTGTTT 3' |
| MHJ-RS01115 | F:5' GAAATACTTATGGCATTATCGGC 3'  R:5'AGATATGAATAGAGCCTGAACTTGG 3' |
| MHJ-RS01130 | F:5'TCTTACAATTCTAATCGGTGTTCCC 3'  R:5' GCAATCGCTGGAAGGACAAA 3' |
| MHJ-RS01615 | F:5' AAAGTGAAAGTCGCGTTGCT 3'  R:5' TTCGCTTACGTTTAAGGTGTCTAA 3' |
| MHJ-RS01655 | F:5'TAGAAATACAACAAGCCGCTAATTC 3'  R:5' TTTCATGGTCCAAATTACCAGTT 3' |
| MHJ-RS01660 | F:5' GGAACAACAGGCTAAATACGTGT 3'  R:5' TCTTGAAAGCGAACGGAGAA 3' |
| MHJ-RS01805 | F:5' GTCTTTGTCGTTGGACCTTCTG 3'  R:5' AATATTTTCTATGCCAGTAGCAGATT 3' |
| MHJ-RS01970 | F:5' CCGTGGAATCGTCCGTCAT 3'  R:5' GCCTCTTCTTGGTCGTGGGTA 3' |
| MHJ-RS02020 | F:5' GGAAAGTCAAAACAGTGCGATTA 3'  R:5' GACCCTGAAGGCCCTAAAAT 3' |
| MHJ-RS02395 | F:5' GCCCTTGATGAAACTAATACCAAT  3'  R:5'  TTCAAAACTAAGGGATTTATGTCGT  3' |
| MHJ-RS02400 | F:5'ATGTTCAGGAATATGCTAATTTATATTTTT3'  R:5' AAGTTTGTCTTGTACCGATGCC 3' |
| MHJ-RS02660 | F:5' TATTAAAGCCGTTGATGGAATTG 3'  R:5' TTCATAAAGGCGAACAAGACAA 3' |
| MHJ-RS03215 | F:5' ATTGTAGCCGCTGATGGAAC 3'  R:5'ATCTACTGAAGTAATCTCACCTCCAGT 3' |
| MHJ-RS03230 | F:5' ACGGAATTATGATTATTGGAGC 3'  R:5' AGGAGACTGCTGGTGCTAGAATA 3' |
| MHJ-RS03330 | F:5' AACCGAAAACAAAAGATGAGATTAG 3'  R:5' CTTTTGGAGAATAAAGTGAGCGT 3' |
| MHJ-RS03540(MATE) | F:5' ATCAAATGCTAAGTGCGGTTCA 3'  R:5' CATTTGAGATTAGGGTCAGCAAAG 3' |
| 23S rRNA | F:5' CTATCTGATGTGGGCGTTGG 3'  F:5'CTTTGGCAAGACAACTGGAA 3' |
